# Inversion of the Uterus

**Published:** 1882-10

**Authors:** Frank V. Walton, Edward A. MacLellan


### VIII.—INVERSION OF THE UTERUS.

REPORTED BY DRS. FRANK V. WALTON AND EDWARD A. MACLELLAN.

Was first called to see the cow at eight o'clock P. M. At this time was informed that the animal had thrown a calf at eight o'clock A. M., which was followed by a speedy inversion and protrusion of the uterus.

Between eight in the morning and eight in the evening, repeated attempts had been made to reduce the organ, but each proved futile. The uterine body had been laid in a tub of ice water, which had partially controlled the hemorrhage, but the walls of the organ were made quite thick and hard by this process.

When first called in the evening the uterus was cold, hard and quite black, as if about to become gangrenous.

The haunches were raised some two feet from the floor by thick bundles of straw. The projecting uterine body was then washed with warm water containing a small quantity of carbolic acid a solution about 1 to 60. This was kept up for about one-half hour. It caused quite a free hemorrhage from the mucuous surface, the walls of the uterus, however, became gradually warmer, softer and more flexible. Dr. MacLellan then grasped the mass in his hands as well as could be done, and by constant pressure and some kneading, at the end of half an hour, the organ was with great difficulty forced back into its place.

Numerous attempts were made by the cow to expel it, and it was with great difficulty kept in position until a permanent support was applied.

A graduated compress was applied over the vagina and perineal region and held in position by a tea bandage. Just before applying the compress and bandage the uterine cavity was washed out with warm water containing alum and opium, five ounces each to the pint.

The animal was given six ounces of brandy and one ounce of tr. opie per orem. She was now left in charge of the stableman until the next morning.

When seen in the morning, she was found standing and eating at the manger as usual, but it was noticed that she was quite weak. During that day she made a few weak efforts to expel the uterus.

The uterus was again washed out with a weak alum and carbolic acid solution, and the compress again applied.

On the third day, the discharge from the vagina had an offensive odor and contained numerous cheesy flakes.

The compress was now removed for good, as there was no indication of any attempt to further expel the uterus. The cavity of the uterus was then thoroughly washed out with a warm solution of alum and carbolic acid, and left without any support.

On the fifth day after the replacement all discharge had ceased, and the animal was perfectly recovered.

New York City, June, 1882.

[This case is quite interesting, as it shows that the uterus can be returned even after being down for some time, if time and patience are brought to bear upon the case. But with all this, it is no easy matter to return the organ; but if returned, the animal is far more valuable than one in which the protruding mass has been amputated. This is especially so if the animal be a valuable breeder. Long and persistent efforts should be made to restore the organ before attempting an amputation.—Ed.]

